# Using biomaterial-based 3D in vitro cancer models to solve current clinical problems

**DOI:** 10.1038/s41416-026-03392-3

**Published:** 2026-04-09

**Authors:** Eve Tipple, Ellen Slay, Olga Tsigkou, Ananya Choudhury, Julie Gough

**Affiliations:** 1 Department of Materials, Faculty of Science & Engineering, School of Natural Sciences, University of Manchester, Manchester, UK; 2 The Henry Royce Institute, University of Manchester, Manchester, UK; 3 Division of Cancer Sciences, Faculty of Biology, Medicine & Health, University of Manchester, Manchester, UK; 4 Department of Clinical Oncology, The Christie NHS Foundation Trust, Manchester, UK; 5 NIHR Manchester Biomedical Research Centre, Manchester University NHS Foundation Trust, Manchester Academic Health Science Centre, Manchester, UK

**Keywords:** Experimental models of disease, Cancer microenvironment

## Abstract

Recent advances in the field of biomaterials show promise in developing pre-clinical models that could elucidate new and innovative treatments for cancer. Both cellular and acellular components can drive cancer formation, progression, and metastasis. Biomaterial-based 3D in vitro models can mimic both these cellular and acellular components. Highly tuneable and biocompatible materials such as hydrogels provide a scaffold for in vitro investigations, mimicking the tumour extracellular matrix structure, upon which cancer cells and additional cellular components can be seeded. Such models have already shown good mimicry of the tumour microenvironment, demonstrating a platform that can be used for drug screening, investigation of treatment response, and a model for the mechanisms of cancer progression. The limitations of current preclinical models include long development times, false-positive drug screening results in 2D cell culture models, and high cost of animal models. This review aims to show the role of biomaterial-based models in addressing existing clinical problems by bridging the gap between current research outcomes and their potential clinical impact.

## Introduction

Cancer detection and treatment has made great progress in recent years however, despite this, cancer still remains a leading cause of death worldwide [1, 2]. A common bottleneck in advancing cancer treatments can be found in the process of treatment testing and development. For all diseases it is estimated that 90% of drug candidates will fail Phase I clinical trials and for those that do pass encounter, on average, a 12-year-long research and development process costing around £1.15 bn [3]. Prior to drugs entering Phase I clinical trials in vitro models play a key role in the drug development process; therefore, enhancing these models provides an opportunity to improve on the speed and percentage of cancer drugs making it to clinic.

**Fig. 1:**
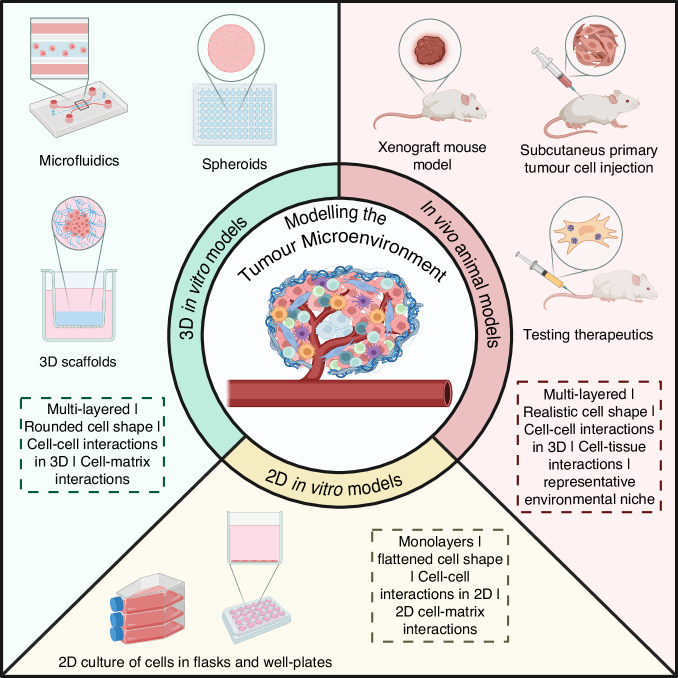
Comparison of methods implemented to model the tumour microenvironment, including in vivo animal models, 3D in vitro models, and 2D in vitro models. Animal models provide an environmental niche that allows cell behaviour like that seen in native tumour. This allows for realistic cell morphologies and behaviour to be seen. Cells that are cultured in 3D exhibit characteristics and behaviours more comparable to the cell characteristics and behaviours seen in vivo than cells cultured in 2D. This includes a more realistic and rounded cell morphology in 3D cultures, compared to the flattened morphologies seen in 2D cultures because 3D cultures also allow cellular interactions in all dimensions, whereas 2D cultures are limited to cellular interactions in two dimensions. 3D models provide an opportunity to incorporate multiple cell types and cell-cell/cell-matrix interactions that cannot be captured in 2D, they not only better mimic the complex nature of the tumour microenvironment but also allow physical changes, such as gradients of oxygen, to be modelled.

Traditional cell culture methods using tissue culture plastic (TCP), often known in biomaterials research as ‘2D cell cultures’, comprising of monolayer cultures on 2D substrates are widely used as basic in vitro cancer models and will be referred to in this review as ‘2D models’ [4]. 2D models probe new cancer treatments using established cell lines [5–8] and have led to significant progress in cancer modelling despite several limitations. These include monolayer formation, limited cell-cell and cell-matrix interactions, and a more ‘flattened’ cell morphology (Fig. 1). Due to the heterogenous nature of native tumour, 2D cell cultures cannot capture many of its important interactions and features such as cell–matrix interactions, oxygen gradients, or the remodelling of the tumour microenvironment [9–11]. Cells cultured on tissue culture plastic lose some original features and functions, such as gene transcription and protein expression levels, cell signalling, and morphology [12]. A solution to these limitations can be found in 3D cultures of cancer cells (Fig. 1). The idea of 3D tumour models better representing native tumour behaviour was suggested in 1994 where differences in behaviours such as function and viability were found between bladder carcinoma cells cultured on TCP and in alginate [13]. More recently, similar differences in function have been demonstrated by comparing 2D and 3D models of cultured human mammary fibroblasts [14]. The improvements made in both in vitro and in vivo cancer models highlight another limitation of traditional 2D models; overestimation of the effectiveness of anti-cancer drugs. Despite promising pharmacological activity in 2D cell cultures, many compounds fail to demonstrate efficacy in vivo, contributing to the low clinical success rate [15–17]. An example of this is demonstrated in investigation of nanoparticle-based cancer therapies and how cells responded to doxorubicin-loaded polymer nanoparticles. Compared to cells grown in 2D, cells grown in 3D expressed higher levels of multidrug resistance proteins, including multidrug resistant protein 1 and lung resistance-related protein, both at protein and mRNA levels [17]. The realisation that 2D models do not completely recapitulate the spatial, cellular, and chemical environment of highly complex tumours and their stroma has meant a shift towards 3D models [18]. Whilst 2D models remain valuable and essential tools for preliminary research, developing 3D in vitro cancer models of cellular and non-cellular components can bridge the gap between 2D models and pre-clinical animal models by providing a more representative view of the in vivo tumour without the high financial and ethical cost. Allowing for more accurate results in applications such as drug screening meaning less false-positive results than in the 2D models commonly used for drug screening. This review discusses the need for greater focus on 3D in vitro models, the developments made in innovative materials used as scaffolds in 3D models, and the translational ability of 3D models in solving problems found in the clinical setting.

## Moving away from in vivo models

Table 1 summarises in vivo and in vitro cancer modelling methods. In vivo models, involving animals and derived tissues, have been traditionally favoured for pre-clinical testing due to their inherent compatibility and natural ability to mimic conditions required for tumour growth. However, conditions provided by animal models fail to capture the negative side effects seen on healthy cells, drug resistance, and immune responses of the tumour. In vitro models using biomaterial-based scaffolds allow for scaffold customisation suiting the needs of the tumour being modelled. This allows for multidimensional cell–cell interactions as well as cell-extracellular matrix (ECM) interactions that are seen in vivo. Fine-tuning the scaffold, upon/into which cells can be seeded, is often a pre-requisite for fabricating a 3D in vitro cancer model. These scaffolds typically need to be optimised to become biomimetic and recreate the natural conditions required for tumour growth. Despite this challenge, there is a drive towards in vitro modelling partly due to the ethical issues surrounding the use of animals in research. This large movement towards replacing, reducing, and refining animal testing, known as the 3Rs has long been part of the UK Animals (Scientific Procedures) Act (ASPA) 1986, and in 2004 the UK National Centre for the 3Rs was established [19]. Moving away from in vivo models will also address the lack of translatability seen in drug screening between 2D in vitro models, animal-based pre-clinical models, and clinical trials. Few clinical trial drugs are approved by regulatory agencies, with oncology drug discovery only leading to clinical trials in approximately 5% of cases, highlighting the inefficiency of the process [20]. A reason for this being that animal models are unpredictable at mimicking human biology and improvements need to be made to justify the large economic and ethical cost of both animal models and failed clinical trials [21, 22]. These ethical issues, economic costs, and scientific limitations are not isolated factors but deeply interconnected and mutually reinforcing. Scientific limitations, such as inaccurate prediction of drug efficacy, directly contribute to economic costs, including the cost of failed clinical trials. Whilst the cost of fabricating biomaterial-based 3D in vitro models is greater than traditional cell culture model methods, the cost of failed clinical trials outweighs this. Biomaterial-based 3D in vitro models offer a new method to streamline the process from research to clinical trials, which would reduce the economic cost long term.

**Table 1:** Comparison between 2D cell cultures, 3D in vitro models, and patient-derived xenografts in terms of their properties when used to create cancer models.

Model Type	Cell characteristics	Available interactions	Tunability	High-throughput?	Cost	Limitations
2D cell cultures	Altered morphologies (‘flattened’), loss of diverse phenotypes.	Cell-cell interactions in two dimensions only, across surface of TCP.	Easy to tune but limited parameters that are available to be altered.	Yes	Low	Does not mimic the structure of tumour and cannot represent all cellular interactions.
3D in vitro models	Morphology more like in vivo, some diversity in phenotype seen.	Cell-cell interactions in three dimensions, cell-matrix interactions with scaffolds, environmental ‘niches’ can be created.	Can be easy to tune with lots of parameters available to tune such as biomaterial selection, material properties, cell types, and cell seeding density.	Yes	Low-medium	Can be difficult to maintain cultures for longer periods of time, difficult to mimic the exact tumour stroma required, and have limited reproducibility.
Patient derived xenografts (in vivo)	Morphology and diversity in phenotypes preserved from in vivo.	Cell-cell interactions in three dimensions, cell-matrix interactions with animal tissues, environmental ‘niches’ occur spontaneously.	Limited tunability once the xenograft has been placed in the animal.	No	High	Large economic and ethical costs associated with animal testing, fails to predict the behaviour of healthy human cells that may surround the tumour, and time consuming.

By addressing scientific limitations, such as recapitulating the TME, through more predictive 3D models, a holistic solution is offered simultaneously reducing economic waste and improving ethical practices. In vitro models can reduce the inflated drug response of 2D culture models, allowing more careful selection of drug targets. Also, a pre-clinical model can be developed recapitulating the in vivo TME, screening potential drugs before use in an animal model. This reduces the number of animals needed for finding new therapeutic targets for cancers. Alongside drug screening, 3D in vitro models also allow the investigation of tumour progression, response to treatments such as radiotherapy, and the unravelling of mechanisms associated with immune responses caused by cancer cells. This interconnectedness strengthens the overall argument for 3D models, positioning them not just as a scientific advancement, but as crucial innovations for creating a more sustainable, efficient, and responsible drug development ecosystem. The driving force behind 3D in vitro model evolution is not simply to create ‘better’ models in general, but to develop more biologically relevant models that recapitulate the intricate spatial, cellular, and chemical environment of in vivo tumours in a way that 2D culture models and patient-derived xenografts cannot (Table 1).

## Mimicking the tumour microenvironment

**Fig. 2:**
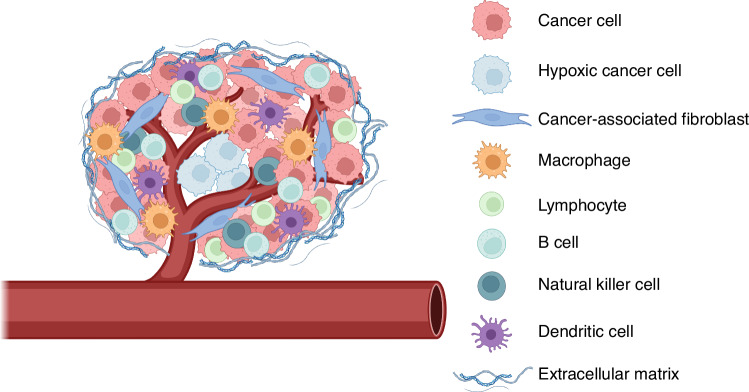
The cells involved in the tumour microenvironment. The tumour microenvironment is complex and dynamic. Each type of tumour will vary in the exact components of its tumour microenvironment however, all tumour microenvironments will generally include the same types of cells [17, 89]. These cell types include cancer cells, fibroblasts (typically cancer-associated fibroblasts), an immune landscape, and adipose cells. Tumours will also include an extracellular matrix that undergoes remodelling by the cancer cells [18, 24].

Tumours are highly heterogeneous systems (Fig. 2) that can be challenging to mimic. In vitro models range from traditional cell cultures (‘2D models’) to 3D spheroid and organoid-based systems. To successfully fabricate an in vitro model, representing the TME, common biological components must be considered (Fig. 2). The exact nature of these components varies depending on the cancer type; however, some are common amongst most types of tumours. These include cancer cells, the ECM, cancer-associated fibroblasts (CAFs), immune landscape, endothelial cells, and adipocytes. Most in vitro models begin with material selection to represent the ECM followed by adding cancer cells. Complexity is added by introducing other cell types into the model, creating a co-culture.

In vivo, the ECM greatly influences the behaviour of cancer cells, with the link between ECM and gene expression realised in 1982 [23]. The ECM provides dynamic, physical support for cells and initiates the biochemical and mechanical signals used in tissue morphogenesis, differentiation and homoeostasis. Tumour ECM components, including collagens, proteoglycans, and glycoproteins are constantly undergoing post-translational modifications by cell-secreted remodelling enzymes which change the topology of the matrix surrounding the cells, therefore influencing cell behaviour [24]. This creates challenges in synthetically mimicking ECM, hence the widespread use of Matrigel^®^ to fabricate in vitro models in cancer research [25–27]. Due to the ethical and financial costs of Matrigel^®^, and batch-to-batch variability, alternative biomaterials are being investigated as scaffolds. The scaffold used to recapitulate the ECM is a key aspect of in vitro model design. A range of biomaterials that have been used as scaffolds to support the growth of cells are shown in Table 2. Selecting an appropriate biomaterial to represent the ECM is challenge for in vitro modelling due to the complexity of the ECM. A benefit of using biomaterial scaffolds is their high degree of flexibility in terms of physical properties, allowing properties such as stiffness to be easily altered and monitored both during the fabrication of the model and in ongoing cell culture. This can be a useful tool as matrix stiffness is considered an important factor in cancer progression [28]. Tumour tissues are typically stiffer than healthy surrounding tissue, for example, breast tumour tissue is $\sim$4 kPa, whereas normal breast tissue is $\sim$0.2 kPa [29]. Techniques commonly used in biomaterials research, such as rheology, can track these changes in stiffness over time [30]. Also, the increase in stiffness due to ECM deposition by cancer cells can be monitored in 3D in vitro models by immunofluorescent or histological techniques, allowing ECM deposition to be quantified and visualised spatially [31–34].

**Table 2:** Examples of 3D in vitro models that allow for the investigation of cancers across varying applications.

Cancer	Cell types	Material used as scaffold	Application
Breast Cancer	Panel of 25 human breast cancer cell lines	Lamin-rich ECM	Investigate morphology of cells when cultured in 3D [36].
	MCF-7 MDA-MB-231	Self-assembling peptide hydrogels	Developing a platform for drug testing [39].
	MCF-7	Alginate hydrogels	To study the role of substrate elasticity on breast cancer cell activity [40].
	MDA-MB-231 HUVECs Adipocytes	Silk-fibrin scaffold and GelMA hydrogel	Development of an anti-cancer drug screening platform [64].
	MCF-7 T47D MDA-MB-231	Matrigel^®^	Used to create a tumour model that would aid in establishing xenografts [26].
Colorectal Cancer	Primary colorectal cancer cells Lung fibroblasts HUVECs Primary natural killer cells	Fibrin gel in a microfluidic system	To develop a model of immune cell infiltration in colorectal cancer [71].
	HT29 HCT116 Cancer-associated fibroblasts	Type I collagen hydrogel	To study the role of cancer-associated fibroblasts in mediating cancer progression and remodelling of the tumour stroma [49].
Gastrointestinal cancers	Cells derived from mouse gastric glands	Hydrogels made from decellularised gastrointestinal tract tissue	To create an alternative to Matrigel for culturing gastrointestinal organoids that can be used as a tumour model [86].
Glioma	Glioma stem cells GSC23	Alginate-fibrinogen hydrogel	To investigate the link between the ITGA2/p-AKT signalling pathway and radiotherapy resistance [79].
Lung Cancer	Murine Lewis Lung Carcinoma cell line NOR-10 murine fibroblasts	Self-assembling peptide hydrogels Agarose gel	To create a model of tumour metastasis [53].
Non-small cell Lung Cancer	A549	Type I collagen hydrogels in a microfluidic system	Development of a platform to use in cell-invasion studies [41].
Oral Cancer	OSCC-3	Poly(lactide-co-glycolide)	To study the effect of microenvironmental conditions on tumour malignancy in vitro and in vivo [87].
	HSC-4 NHDF	Collagen gel matrix	To create a model for clinical radiation therapy testing [77].
Ovarian Cancer	Primary ovarian cancer cells	Peptide amphiphiles	Proof-of-concept study with clinically used chemotherapeutics to validate model [38].
	PEO1 PEO1-cDDPr PEO4 PEO14 OV(hyg)CAR3	Matrigel^®^	Used to create a tumour model that would aid in establishing xenografts [26].
Pancreatic Cancer	MIA PaCa-2 PANC-1	Nanocellulose hydrogels	Screening of anti-cancer treatments [88].
	PANC-1	Polymeric scaffolds	To create a model for radiotherapy treatment screening [78].
Squamous cell carcinoma	PDVA PBMCs	Type I collagen hydrogel	Co-culture to study behaviour of tumour-associated macrophages [47].

Scaffolds, such as hyaluronic acid- and peptide-based hydrogels, are highly tuneable providing more opportunities to modify and tailor the scaffold. Such modifications can allow the scaffolds to better interact with cells and maintain a higher level of viability than the unmodified material. Alternatively, scaffolds derived from natural materials can be used which are typically better biomimics and often more biocompatible than synthetic scaffolds; however, they can lack the required physical properties needed to mimic tumour tissue stiffness. Therefore, tuning the biomaterial to suit the needs of a model can become a balancing act of biocompatibility and physical properties. Some scaffolds are created that focus on one key ECM component, for example malignant breast epithelial cells have been cultured within a laminin-rich biomaterial matrix [35]. The morphology and gene expression of 25 epithelial cancer cell lines were studied, reporting the first large-scale comparison of the transcriptional profiles and 3D culture phenotypes of these cell lines [36]. However, both studies relied on basement membrane obtained from Matrigel^®^ which, alongside the discussed limitations, has non-tissue-specific ECM [37]. More recent studies have used modified, synthetic hydrogels such as peptide hydrogels to mimic the nanofibrous architecture of ECM. Peptide hydrogels contain biocompatible peptide sequences, such as those for collagen or laminin. A peptide-based 3D matrix has been developed to study ovarian cancer [38], fabricated using peptide amphiphiles, which self-assemble forming nanofibres, and display cell-instructive motifs mimicking the function of ECM proteins such as laminin, collagens, and fibronectin. The ovarian cancer cells formed tumour spheroids and behaved similarly to when seeded in Matrigel^®^ and observed in vivo. Self-assembling peptide hydrogels (SAPHs) have been used in a model of breast cancer where the mechanical control provided by the peptide sequence could better mimic the stiffness of breast tumour tissue than Matrigel^®^ or collagen I gel. The SAPH could also recapitulate key features of solid tumours such as hypoxia and invasion and was shown to act as a barrier to drug delivery, having drug penetration similar to that seen in vivo [39]. Alginate hydrogels have also been explored for fabricating in vitro models for breast cancer where the cellular response to matrix stiffness was investigated using MCF-7 cells seeded into alginate hydrogels of varying stiffness [40]. Expected morphological changes were found between 2D and 3D cultures, including a flat cell morphology forming monolayers in 2D versus a more rounded morphology and cluster formation in 3D. As stiffness increased, proliferation decreased but did not alter their cluster-forming ability. This highlights the importance of biomaterial selection to accurately represent the physiological environment of the tissue being studied. Similar morphological changes were seen when culturing the non-small cell lung carcinoma cell line A549 in collagen I gels. These cells exhibited more rounded morphology when seeded into collagen I gels than cells seeded on TCP [41].

The tunability of biomaterials extends beyond initial fabrication and should include the capacity for dynamic changes over time, or ability to actively respond to and be remodelled by cellular cues. Biomaterials can be modified in use to allow dynamic modelling of the TME through stimulus response, such as light, enzymes, or the use of adaptive materials, for example, with supramolecular reversible linkages that are force-responsive [42–44]. This represents a more challenging level of biomimicry and presents a future direction for biomaterial research: developing smart, responsive, or remodelling-capable biomaterials that can actively adapt their properties in response to cellular activity and disease progression. Such advancements would further close the gap between in vitro models and the complex, evolving conditions seen in vivo, enhancing their predictive power for long-term studies of cancer progression and treatment resistance.

## Adding layers of complexity

To improve the clinical translation of in vitro models, the challenging task of increasing their complexity must be undertaken. Alongside cancer cells, the TME contains many cell types (Fig. 2) which can be added to the model, allowing for the investigation of a variety of cellular interactions and responses. In addition, the use of in vitro models allows for the incorporation of human immune cells, rather than those native to the animal models, further enhancing opportunities for clinical translation. A limiting factor for increasing the complexity of an in vitro model to better mimic a native tumour is the time frame that is feasible for an in vitro culture of cells. In patients, tumour development can range from weeks to years depending on the type of cancer and the patient. Maintaining a 3D in vitro model for this length of time is difficult, and few reports culture such models past 21 days. Despite this 3D in vitro models have shown to be useful platforms to investigate smaller scale cellular interactions such as those between tumour cells and immune cells, and for investigating the progression of cancers in a controlled environment [45, 46]. For example, in the presence of squamous cell carcinoma cells, cultured in collagen I gels, human-derived macrophages become activated, with polarisation towards M1 and M2 phenotypes [47]. Tumour cells have also been cultured in 3D with fibroblasts to investigate metastasis and invasion in lung, breast, and colorectal cancer [14, 48–51]. Progression this field was shown by a co-culture of pancreatic tumour cells and fibroblasts, using Matrigel^®^-coated invasion chambers and soft agar colony formation, to investigate the effects of fibroblasts on pancreatic cancer cells [50]. In this case the metastatic behaviour of the tumour cells in the presence of fibroblasts was only able to be validated in vivo because the 3D model did not allow for compartmentalisation. This improved in 2020 with a co-culture of colorectal cells with CAFs in a 3D collagen gel, which allowed the model to be compartmentalised with tumour cells in the centre and CAFs surrounding the outside of the gel [49]. The presence of CAFs on the outside caused the tumour cells to have an increased distance and surface areas of invasion into the collagen gel, towards the CAFs. Additionally, the tumour cells showed matrix remodelling of the collagen gel through the disruption of endothelial networks and upregulation of vascular endothelial growth factor. These more realistic and complex models have enabled discussions around the use of in vitro models as pre-clinical tools due to their ability to mimic detailed aspects of the complex TME and cellular interactions.

## Solving current clinical problems

Current inefficiencies in drug discovery, where preclinical research often fails to reliably translate to clinical success, cause financial losses, ethical concerns, and ineffective treatments. The fundamental reason for the shift to 3D models is to overcome the bottleneck in drug discovery and patient care, fostering a more efficient and responsible ecosystem for developing new therapies. The increasing complexity of in vitro cancer modelling through co-cultures and more complex matrices discussed above allows more relevant pre-clinical models to be established. These models have several applications making them powerful tools in modelling tumour progression, to helping elucidate drug targets, biomarkers, and immunotherapy routes. Table 2 summarises 3D in vitro models currently being explored.

## Modelling mechanisms of tumour progression

The more complex 3D in vitro models can provide contextual and chemical cues, such as multidimensional cell interactions, mechanical, and biochemical support from the scaffold. These cues are essential to cellular processes such as metabolic activity, morphology, and differentiation [52]. This makes 3D in vitro models viable platforms for investigating tumour metastasis and progression.

SAPHs modified with peptides such as arginine-lysine-aspartate, derived from fibronectin, have been utilised as scaffolds for spheroids containing Murine Lewis Lung Carcinoma (LLC) cells and murine skeletal muscle fibroblast (NOR-10) cells [53]. The interaction of the cells with the modified SAPH enhanced the metabolic activity of the spheroids, promoting the invasiveness of the LLCs via vinculin expression. Cell migration was enhanced through an induced epithelial to mesenchymal transition, seen in some epithelial cancers where cells switch from an adherent phenotype to a migratory, metastatic mesenchymal phenotype. Whilst this is an effective example of in vitro modelling of tumour progression; it is important to determine if the same cell behaviours are seen with human cell lines and primary human cells.

Alongside promoting signalling pathways, 3D cultures also encourage cancer cells to deposit ECM, demonstrating another in vivo behaviour, remodelling their TME [54, 55]. This has been demonstrated in a 3D culture using a commercial SAPH seeded with human breast cancer cell lines MCF-7 and MDA-MB-231 [39]. These cells produced proteins commonly found in the ECM such as Collagen I. This also highlighted another example of how in vitro models can be used to show tumour progression by modelling hypoxia [10]. In vivo tumours initially rely on diffusion of oxygen and glucose for their metabolism; however, tumours will rapidly outgrow the diffusion limit, resulting in chronic hypoxia. This then induces angiogenesis, through HIF dependent activation of pro-angiogenic genes, a hallmark of cancer [56, 57]. The SAPH and MCF-7/MDA-MB231 breast cancer model showed the presence of some positively stained cells for HIF-1α, a transcription factor that aids in adaption to a low oxygen ( > 5%) environment, after 1 day in culture and increased in prevalence by day 14, suggesting this model recreates hypoxia seen within solid tumours. By clarifying exact mechanisms of tumour progression, new targets for drug treatments can be found. In vitro modelling enables quicker progress in this field by utilising high-throughput drug screening, as in vitro models are faster and cheaper to produce than in vivo testing.

## High-throughput drug screening

Multiple factors result in the inefficiency seen in the drug discovery process, from identifying a drug target to a successful clinical trial [20]. 3D in vitro models offer an improved platform to assess drug efficacy and toxicity than 2D cell culture models. Novel examples of ‘body-on-a-chip’ microfluidic platforms explore therapeutic responses on a range of on target and off-target organs. Often, 2D platforms give an inflated drug response, allowing the drug target to progress into pre-clinical development [58]. The in vivo mouse model is commonly used in pre-clinical development and patient-derived xenografts can be transplanted (patient tumour biopsy) into immunocompromised mice [20] however, is time-consuming and costly. It is within pre-clinical development that in vitro models could become a powerful tool, not only reducing the number of animals needed (reducing ethical concerns and cost), but would also have better prediction of drug efficacy, toxicity, and immune system interaction. 3D in vitro models provide a better representation of the TME than 2D models as they allow architectural arrangements of cells that better mimic cell-cell and cell-matrix interactions. Together, these steps in developing more complex 3D models allows for an improved prediction of drug efficacy and toxicity.

There are reports of in vitro models being used to investigate the response of cancer cells to commonly used anti-cancer drugs, which can be scaled up to offer high-throughput drug screening platforms [17, 59–63]. These include breast and ovarian cancer cell responses to tamoxifen, doxorubicin, and cisplatin using peptide and GelMA hydrogels, demonstrating the potential of in vitro models in replacing in vivo models in some pre-clinical testing [39, 64, 65]. 3D cultures have also shown a delayed period between the administration of tamoxifen and the response of the cancer cells, which is more representative of in vivo culture than seen in 2D models [39]. A co-culture model of MDA-MB-231 with adipocytes and HUVECs in GelMA has also shown promise in drug screening capabilities, mimicking features observed in vivo, such as cross-talk between cell types, drug penetration, and anticancer drug resistance to doxorubicin and cisplatin [64]. Whilst in vitro models have proven to be promising candidates for pre-clinical screening, challenges remain. One such challenge is the reproducibility of in vitro models. The scaffold can sometimes exhibit batch-to-batch variability when synthesised or manufactured. One example of this is Matrigel^®^, where there is little consistency in physical properties, such as stiffness, between batches [37]. This would make it difficult to obtain a pre-clinical model that would obtain consistent results. Additionally, variations in culture conditions between laboratories, such as the frequency of media replenishment, can cause inconsistencies. To create repeatable in vitro pre-clinical models for widespread use, strict guidelines are required. Despite these challenges, biomaterial-based 3D models have immense potential as an alternative to traditional drug screening and as a means of reducing animal use in pre-clinical models.

## Investigating treatment response

The progression of tumours in both immunodeficiency and inflammatory responses has been of key interest in developing immunotherapeutic approaches. Two main immunotherapeutic approaches have emerged: immune checkpoint inhibitors (ICI) and adoptive T cell therapy (ACT). ICI therapy targeting PD1/PD-L1 and CTLA-4 has been used clinically for cancers, including small cell lung cancer, renal cell carcinoma, and squamous cell skin cancer [66–68]. However, due to adverse side effects and high financial cost, there is a careful process for patient selection and personalisation. ACT has been successful in CAR-T cells targeting CD19 in B cell lymphoma and lymphocytic leukaemia, there have been challenges in breaking through into solid tumours due to the complex TME, antigen heterogeneity, and difficulty of CAR-T cells reaching the tumour sites [69]. ICI and ACT have become important tools clinically; however, to overcome challenges and improve clinical translation of these therapies, research on the tumour immune microenvironment is needed. A common method of achieving this is 2D co-cultures of tumour cells and exogenous immune cells, straightforward and cost-effective methods of investigation, however, as seen with 2D models for drug discovery, they do not accurately represent the in vivo immune landscape as 2D environments lack the spatial and temporal aspects seen in vivo. 3D in vitro models could simulate the 3D in vivo structures spatially, therefore reproducing some of the physiological characteristics of the native tissues.

3D in vitro co-culture models allow human-derived immune cells to be incorporated into co-culture models, as shown in an in vitro model of pancreatic cancer organoids cultured in Matrigel^®^ [70]. When T cells, derived from peripheral blood mononuclear cells, were added to the cell culture medium, they migrated towards the pancreatic organoids, through the Matrigel^®^, showing a migratory response of immune cells. 3D in vitro models have been developed that recreate some of the spatial aspects seen in vivo. Hydrogel scaffolds have been used to fabricate vascularised networks needed for colorectal cells where natural killer (NK) cells could be introduced [71]. The in vitro nature of the model allowed NK cell activity to be monitored using live-cell imaging with migration and cytotoxic activity of NK cells against six different colorectal cancer cell lines tested, highlighting the high-throughput testing available in in vitro models. There are several examples in the literature of patient-derived organoids in muscle-invasive bladder cancer, ovarian cancer, and neuroblastoma [72–74], which can be used in pre-clinical trials of immunotherapeutic approaches; however, these organoids are patient dependent. In vitro organoids with biomaterial scaffolds are grown in a more controlled environment making consistency easier to achieve than with patient-derived organoids. Once a reliable 3D in vitro testing platform is established, the element of personalisation can then take place with tumour cells isolated from patients. The use of human-derived cells and tissues raises important ethical challenges. Historical examples such as the HeLa cell line highlight issues surrounding informed consent and donor autonomy, while the increasing use of patient-derived cells introduces, further considerations, including data privacy, long-term storage, and potential secondary or commercial use of biological material [75]. Although strengthened informed consent frameworks and ethical review processes have mitigated many of these concerns, ongoing ethical oversight remains essential in the development and application of human-derived 3D in vitro models [76].

Radiotherapy is widely used for many cancers, however, there are challenges faced in the clinic with damage to healthy tissue surrounding the tumour and resistance developing in tumours, typically those with high levels of hypoxia. Radiation-induced damage is only directly assessed by biopsy which is often too invasive to be done routinely. Radiation of head and neck cancers often causes damage to surrounding tissues, resulting in ulceration and necrosis. An in vitro model would allow methods of administering radiation to be tested, monitoring the impact on normal cells, which could not be accurately studied using a 2D model, due to the complex architecture of the tumour. This was shown in a model containing human tongue squamous carcinoma cells (HSC-4) and fibroblasts, in a collagen gel [77]. The radiobiological efficacy of HSC-4s and fibroblasts was evaluated in 2D and 3D collagen gels, using histological techniques, to assess the tumour cells and surrounding tissues. This type of 3D model has potential as a clinical prediction tool; however, it needs refinement in terms of the exact scale needed to represent the desired tissues and inclusion of other TME components that may influence the radiation response such as ECM components. A recent model for pancreatic cancer addresses some of these limitations by including ECM proteins [78]. The pancreatic cancer cells (PANC-1) were seeded into a 3D microporous polyurethane scaffold surface modified with ECM protein, fibronectin to investigate the effects of hypoxia on radiotherapy resistance. The models were exposed to radiation, and the behaviour of PANC-1 cells monitored by HIF-1α, a marker indicating hypoxia, secretion for up to 7 days after exposure. Post-radiation, the PANC-1 cells had increased viability, ECM deposition, and HIF-1α secretion whilst also exhibiting reduced apoptosis in the hypoxic samples. This model demonstrates the radiotherapy-resistant behaviour seen in vivo, revealing hypoxia-induced radioprotection. Development of such models begins to unravel the mechanism associated with hypoxia, which will allow more targeted treatments to be developed in response to radiotherapy resistance [79].

## Future directions

The utilisation of biomaterials in 3D in vitro modelling of tumours allows the fabrication of a variety of cancer models, including lung, breast, ovarian, bladder, and colorectal cancers [50, 64, 80–83]. These 3D in vitro models have several advantages over traditional 2D cell culture models, specifically the inclusion of the complex and dynamic 3D environment. This is due to advances made in the field of biomaterials, with many hydrogels being chemically modified to better represent the tumour stroma structurally and biologically. Biologically relevant scaffolds have been harnessed to create 3D in vitro models with increasing layers of complexity, including co-cultures, compartmentalised models, and scaffolds with increased ECM deposition.

In vitro models have merged as potential tools to bridge the gap between basic and clinical research in drug discovery, immunotherapeutic approaches, and radiotherapy. Once a reliable library of platforms has been established, in vitro models could allow for high-throughput testing that can improve the efficiency of drug screening and measure the effectiveness of treatment responses prior to clinical trial. This would help streamline the treatment testing processes, reducing the number of animals used in testing in turn reducing the associated ethical and financial costs. As discussed, there are still ethical implications of utilising human-derived cells, especially in personalised models, however obtaining patient consent has become essential and would not outweigh the benefit to the patient should the outcome be a personalised therapy that better treats the patient.

Despite these advances, significant challenges remain in 3D in vitro modelling, such as variability in cell culture conditions (e.g. culture medium composition) which hinders standardisation of protocols. The absence of strict guidelines or standard operating procedure currently limits their applicability as pre-clinical tools. It also remains challenging to maintain in vitro models long term, to timescales comparable to tumour development, making it difficult to achieve the desired levels of complexity in 3D in vitro models. Additionally, some cancers are inherently more difficult to model than others. Cancers which do not form solid tumours, such as lymphocytic leukaemia, have TMEs that are more challenging to recapitulate in vitro than cancers that form solid tumours [84]. Even cancers that form solid tumours can pose greater challenges than others, for example, prostate cancers have proven to be difficult to propagate in the laboratory environment, whilst breast cancers does not face this challenge [85]. The reasons for the variation in modelling difficulty across cancer types are numerous and complex, it is best viewed as a case-by-case basis which is beyond the scope of this review.

In vitro modelling has emerged as a potential tool to bridge the gap between basic and clinical research in drug discovery, immunotherapeutic approaches, and radiotherapy. Once a reliable library of platforms has been established, in vitro models allow for high-throughput testing that can improve the efficiency of drug screening and measuring the effectiveness of treatment responses. The controlled laboratory-based environment also allows for the possibility of reliable, personalised models to be developed that could lead to immunotherapeutic approaches targeted to a specific patient. The heterogeneous nature of cancer would mean that an in vitro model that could be personalised would have a great impact on investigating treatment response.
